# The Role of Agricultural Systems in Teaching Kitchens: An Integrative Review and Thoughts for the Future

**DOI:** 10.3390/nu15184045

**Published:** 2023-09-18

**Authors:** Alexis Cole, Jennifer Pethan, Jason Evans

**Affiliations:** 1 Gerald J. and Dorothy R. Friedman School of Nutrition Science and Policy, Tufts University, Boston, MA 02111, USA; jennifer.litzau@tufts.edu; 2 College of Food Innovation and Technology, Johnson and Wales University, Providence, RI 02903, USA; jason.evans@jwu.edu

**Keywords:** local agriculture, teaching kitchen, culinary medicine, nutrition security, food is medicine, urban agriculture, public health, food systems, planetary health

## Abstract

Diet-related chronic disease is a public health epidemic in the United States. Concurrently, conventional agricultural and food production methods deplete the nutritional content of many foods, sever connections between people and the origin of their food, and play a significant role in climate change. Paradoxically, despite an abundance of available food in the US, many households are unable to afford or attain a healthful diet. The linkages between agriculture, health, and nutrition are undeniable, yet conventional agriculture and healthcare systems tend to operate in silos, compounding these pressing challenges. Operating teaching kitchens in collaboration with local agriculture, including farms, community gardens, vertical farms, and urban agriculture, has the potential to catalyze a movement that emphasizes the role of the food system in promoting human and planetary health, building resilient communities, and encouraging cross-disciplinary collaboration. This paper reviews the current state of agricultural systems, food is medicine, consumer behavior, and the roles within these sectors. This is followed by a series of case studies that fill the gaps between TKs and agriculture. The authors summarize opportunities to combine the knowledge and resources of teaching kitchens and agriculture programs, as well as challenges that may arise along the way.

## 1. Introduction

The prevalence of diet-related chronic disease is at an all-time high in the United States (US). Six in ten adults are living with at least one chronic disease, and this is projected to continue to rise [1]. Simultaneously, conventional agricultural practices and our industrialized food system strips food of much of its nutritional value, disconnects people from the origin of their food, and is a major contributor to climate change. Further, in a country that produces an abundant quantity of food, approximately one in ten US households cannot afford and/or access a nutritious diet [2].

### 1.1. The Conventional Approach

Beginning in the late 19th century, the rapid adoption of new technologies prompted a shift in US agricultural production from small, diversified operations to large-scale, intensive practices, including a reliance on chemical inputs and fossil fuels. As intensification became more widespread, the pervasive trend of consolidation of farmland across the country led to the loss of many small- and medium-sized farms, with the greatest burden being felt by underrepresented groups [3]. Government policies began to favor large scale producers, encouraging farmers to “get big or get out”, leading to a significant decline in diversity among crops, livestock, and producers [4]. What was once a food system that provided communities with nourishing whole foods is now a system that largely prioritizes maximized yields and food processing characteristics, such as shelf stability, uniformity, and multiple uses in food processing [4,5]. While modern agriculture successfully produces more than enough food to fill our plates, it does so at the expense of nutrient density, food quality, and environmental sustainability.

Urbanization in the US marked the beginning of an altered relationship between food and farming. While it brought about many benefits, people became increasingly disconnected from food production. With industrialization and globalization occurring rapidly within the agrifood system, not only did people become less aware of where food came from, but the quality was no longer the same. The development of hyperpalatable food brought about a shift in consumer behavior and diets toward greater consumption of ultra-processed foods (UPFs) [6]. UPFs contain additives such as artificial colors, flavors, preservatives, and sweeteners, alongside salt, sugar, and fat, to improve shelf life, texture, and taste. These products are typically mass-produced with low-cost ingredients, heavily advertised, and designed to enhance palatability and increase consumption [7]. Such food items have become increasingly cheap and accessible, while fresh, healthy food has become more difficult to obtain. As a result, UPFs became a larger part of the diet, contributing 25–57% of daily calories for the average American adult in 2018 [8]. It is well-established that high consumption of UPFs is a major contributor to poor health outcomes. Even within healthcare settings, wherein one might expect to find the most health-promoting foods, nutrient-poor UPFs may be more readily available than nutrient-dense whole foods [9].

With undeniable evidence of the association between the Standard American Diet and poor health outcomes, communities are revitalizing local agricultural systems as one way to promote public health. Local food networks allow consumers to directly access community gardens, urban or rural farms, farming cooperatives, farm-to-school programs, and farmer’s markets [10]. It is well recognized that neighborhoods with limited access to affordable, healthy food (i.e., food insecurity) experience poorer health outcomes than those with greater access [11,12,13,14,15]. Therefore, promoting local agriculture may provide a more effective and sustainable method to address food access inequality by supporting organizations that provide health-promoting foods to underserved communities [10].

Agriculture, health, and nutrition are deeply intertwined, yet there is an evident disconnect between conventional agriculture and healthcare systems. Should stakeholders within these systems continue “business as usual”, they will miss important opportunities to mitigate climate change, reduce the burden of chronic disease, and co-create equitable solutions for a more just world.

### 1.2. The New Perspective

Now is the time to develop novel solutions to reimagine a future in which agriculture and healthcare systems work together synergistically. An emerging field of research illustrating the links between human and planetary health provides compelling evidence that agricultural and healthcare systems can be leveraged to mitigate the climate crisis while improving individual and community health. Healthcare settings offer an opportunity to utilize their purchasing power, frequent patient contact, and position within the community to support the transformation of food systems. Teaching Kitchens are one modality by which physicians, dietitians, health coaches, chefs, community members, and others can apply the concepts of nutrition, sustainability, and preventative healthcare through hands-on culinary training and education.

In this paper, we aim to make the case that operating teaching kitchens (TKs) in collaboration with local agriculture, including farms, school and community gardens, vertical farms, and urban agriculture, has the potential to catalyze a movement that emphasizes the role of the food system in promoting human and planetary health, building resilient communities, and encouraging cross-disciplinary collaboration.

The paper begins with an extensive background review of the current state of agricultural systems, food is medicine, consumer behavior, and roles within these sectors. This is followed by a series of case studies on organizations that are already filling the gaps between TKs and agriculture. Authors conclude the paper by emphasizing the great deal of possibilities to enrich the Food is Medicine movement through the use of TKs and local agriculture, identifying opportunities to continue growing the movement and listing challenges that may arise in doing so.

## 2. Background

### 2.1. Functions and Dysfunctions of the Current Food System

It is unequivocal that the health of people and the planet are inseparable. The current global food system is a major driving force for both climate change and diets that foster communicable and non-communicable diseases [16]. The global industrial food system prioritizes imported foods over local production, significantly influencing dietary patterns. This system—typically characterized by inexpensive, convenient, shelf-stable UPFs—blunts individuals’ connection to regional, seasonal, and indigenous food systems and their consumption of nutrient-rich foods. High consumption of UPFs, particularly fast food in the US, is linked to weight gain and insulin resistance, increasing the risk of obesity and type 2 diabetes [17]. However, studies reveal that greater access to local food markets correlates with lower obesity and diabetes rates [18].

The pervasiveness of chronic disease has been associated with the shift from locally grown agricultural foods to a modernized, import-reliant diet. In the early 20th century, 90% of the land in Puerto Rico was devoted to agrarian activities, which provided the majority of the country’s food supply. Rural areas had lower obesity rates and nutrient deficiencies compared to urban and affluent areas due to their consumption of homegrown starchy vegetables. However, industrialization and tourism policies in the mid-20th century led to decreased local food production, resulting in over 85% of the food supply being imported and dominated by UPFs such as sugary beverages, sweets, and processed meats. This shift diminished diet quality and led to an increase in diet-related disease. In 2017, 17.2% of Puerto Rican adults had a diagnosis of type 2 diabetes—the highest recorded prevalence of diabetes among all US states and territories at the time. Compared to the US, Puerto Rico also has a higher prevalence of hypertension and obesity, which are risk factors for diabetes. In order to demonstrate the relationship between local food consumption and health outcomes, researchers analyzed the association between local food purchasing and diet quality. They discovered that those who purchased local food products had diets higher in fruits, vegetables, whole grains, nuts, and legumes and better met nutritional recommendations. The authors concluded that increased local food production plays a role in improving health outcomes as well as encouraging climate-resilient food systems through increasing plant-based food consumption and the adoption of climate-adaptive farming practices [19]. Local food networks offer a resilient alternative to global food networks, especially in the face of increasing climate extremes and uncertainties [20,21].

#### Agricultural Practices’ Influence on Nutrient Density

Many studies have identified that the nutrient density of food has declined significantly in the last 50–70 years [22]. This is a cause for concern because it is now more challenging to obtain the same amount of nutrients from food as it was in previous generations. This phenomenon has been labeled as “hidden hunger”, wherein an individual is micronutrient deficient despite adequate daily caloric intake, as a result of consuming an energy-dense but nutrient-poor diet [4,23,24]. The consequences of this can be devastating; it is estimated that hidden hunger affects one in three people around the globe [23].

With political and financial pressure to emphasize yield and processing characteristics in conventional agriculture, the quality of food we consume has been declining. Since the “green revolution” in the 1960s and 1970s, grain yields have increased by at least two- to threefold in major developing countries [22]. While grain yields increased, their protein content declined significantly. Studies analyzing protein concentrations of wheat, rice, and barley saw protein levels drop by as much as 30%, 18%, and 50%, respectively [22,25,26]. In short, nutrient composition and flavor, which have a strong influence on palatability and satiation, are being sacrificed for efficiency. 

Climate change is also threatening the nutrient composition of crops. In an assessment of nutrient density in crops under elevated carbon dioxide levels, the overall concentration of 25 minerals in plants, including calcium, potassium, zinc, and iron, was reduced by 8% on average [27]. Similar studies reveal increased ratios of carbohydrates to minerals, as well as lower protein concentrations [27,28,29]. Such declines in nutrient density pose threats to overall nutrient intake and quality and present even greater threats to populations with limited access to fresh produce, whose intake of nutrient-dense foods may already be compromised.

Only one in ten US adults consumes the recommended daily intake of fruits and vegetables [30]. Low fruit and vegetable consumption, coupled with reduced nutrient density, threatens the ability to reach adequate levels of vitamins, minerals, and bioactive compounds essential for optimal health. Even with large quantities of food available year-round in developed countries, inadequate micronutrient intakes appear to be widespread. A recent analysis of publicly available dietary surveys showed that even in affluent countries, more than three quarters of the adult population does not achieve the recommended intakes for a significant number of vitamins [31].

One major contributing factor to this phenomenon is the high prevalence of UPFs in industrialized countries. UPFs–which are high in salt, oil, and sugar, and low in fiber, vitamins, minerals, and phytonutrients–are widely available from the supermarket to hospitals and now make up 73% of the US food supply [32,33]. Epidemiological studies have documented significant associations between greater UPF consumption and diseases such as obesity, heart disease, type two diabetes mellitus, cancer, and depression [33]. A controlled metabolic trial in 20 adults found that increased availability and consumption of UPFs was associated with increased caloric intake and obesity prevalence [34]. As UPFs continue to make up a larger percentage of many Americans’ diets, the likelihood of meeting one’s recommended daily micronutrient intake is reduced. These micronutrients and compounds play a critical role in preventing disease; low micronutrient intake is a contributor to many major illnesses [31,35,36].

However, certain farming practices have been shown to increase the nutrient content of foods. In healthy soil, microbes in the vicinity of plant roots increase the availability of the minerals and trace elements required to maintain the health and vitality of their plant hosts. Practices that prioritize soil health have demonstrated higher levels of vitamins, minerals, protein, omega-3 fatty acids, and phytochemicals in crops. A study comparing the nutrient density of crops grown on conventional farms versus regenerative farms (i.e., those that prioritize soil health and carbon sequestration) found an increase of 50% more zinc and magnesium, almost 50% more carotenoids, and 60–70% more total phenolics in the farms that prioritize soil health [37]. Such gains in nutrient density showcase that climate-friendly farming practices serve as the foundation for the food is medicine movement.

Conventional farming practices disrupt soil biology. As a result, nutrient uptake in plants is significantly reduced, negatively impacting food quality. Additionally, post-harvest practices such as storage, processing, and refinement further alter and reduce the nutrient composition of food. In an assessment of the impact of storage time and temperature on glucosinate levels in broccoli florets, it was found that levels were reduced by 81% after 5 days at 2 degrees Celsius [36]. Glucosinates are sulfur-containing compounds found in cruciferous vegetables that have been associated with disease prevention through anti-inflammatory and antioxidant activities [38]. These findings suggest that sourcing food from local farms that prioritize soil health and local distribution channels rather than distant conventional operations can positively impact nutrient intake and public health outcomes, while also reducing transportation emissions.

Local and regional food systems enhance community vitality by retaining food spending locally and fostering new business prospects. Producers and consumers establish transparent relationships, supporting awareness of food origins and production methods. Strong local food ties bolster resilience against global food supply challenges. Local and regional food systems share the following objectives: boosting local economies, increasing fresh food access, and creating market pathways for beginner farmers and smaller producers [39]. Local and regional agriculture may include traditional farms—both urban and rural, as well as vertical farms, hydroponic farms, and school and community gardens. Small, local farms and gardens (even if not certified organic) tend to be more favorable than large scale organic farms because on average, the latter uses fewer agroecological practices and more closely resembles conventional farming methods, rather than fully embracing diverse, sustainable practices [40].

The EAT-Lancet Commission put forth the concept of planetary health diets, which support human health and prioritize environmental longevity and vitality [16]. Planetary health diets are characterized by calorically-balanced diets containing high amounts of plant-based foods and low amounts of animal foods. These diets are a part of the broader “Great Food Transformation”, which seeks to promote agricultural biodiversity, significantly reduce global food loss and waste, and improve agricultural efficiency and sustainability regarding land, water, and fertilizer use. This proposed transformation offers a solution to counter the potentially detrimental consequences that could arise from persisting with current disease-fostering dietary habits and conventional agricultural practices. The growing awareness of these linkages is attracting global interest in research, education, advocacy, and activism at the intersection of food, nutrition, health, and sustainability.

One potential solution for addressing the concurrent challenges of poor global health and climate change lies at the intersection of kitchens and agriculture, which is not a novel concept to chefs and culinary experts familiar with the farm-to-table movement. However, the relationship between TKs and agriculture and their synergistic effects on behavior change, health outcomes, and environmental sustainability is underexplored in the academic literature. It is at this intersection that the potential to utilize food as medicine, uplift and bring dignity to farmers, highlight the role of the chef, build bridges across often siloed sectors, improve nutrition security, promote health equity, and support environmental sustainability exist. Public- and private-sector organizational leaders have an opportunity and responsibility to facilitate a shift in peoples’ diets to promote individual and planetary health and longevity.

### 2.2. Food Is Medicine, Produce Prescription Programs, and Teaching Kitchens

#### 2.2.1. Food Is Medicine

Food is Medicine (FIM) is a clinical intervention that uses food to help treat or manage disease, promote health, and often improve nutrition security. As a concept, FIM has existed for millennia, in practices such as Ayurveda, traditional Chinese medicine, and Indigenous foodways. However, its application specifically as a clinical intervention in medicine and research has been gaining momentum over the last 20 years. It is most often facilitated through the healthcare system and funded by philanthropy, government, healthcare, or a combination [41]. Varying degrees of FIM interventions may be deployed, depending on a healthcare system’s resources and/or patients’ needs. Mozaffarian et al. developed the Food is Medicine pyramid, which displays a continuum of FIM interventions ranging from prevention at the base to treatment at the top [42]. The pyramid base starts with population-level healthy food policies and programs, followed by government nutrition security programs, produce prescription programs, medically tailored food packages, and medically tailored meal programs. Nutrition counseling and education are recommended for the top four interventions.

The 2022 White House Conference on Hunger, Nutrition, and Health brought FIM into the public dialogue. Organizations such as the Rockefeller Foundation, American Heart Association, American College of Lifestyle Medicine, Community Servings, and more made commitments to fund FIM programs regionally and nationally [43]. This conference and these commitments served as a litmus test for the growing interest in and recognition that FIM can be part of the solution in improving individuals’ health, social determinants of health, and health equity.

#### 2.2.2. Produce Prescription Programs

Produce prescription programs (PPPs) are identified as one of the five food-based nutrition programs or interventions within the Food is Medicine pyramid. PPPs typically provide patients with fruits and vegetables (and sometimes beans, nuts, whole grains, dairy, and eggs) in order to address diet-related chronic diseases and/or food or nutrition insecurity. Patients may also receive nutrition and/or culinary education in addition to food [42]. PPPs may lead to increased fruits and vegetable consumption, improved biochemical and anthropometric measures, and potential healthcare savings amounting to millions of dollars [44]. Adhering to the concept of social prescribing, wherein health professionals direct patients to local resources in order to enhance their health and well-being, PPPs establish connections between patients and community assets for promoting healthy eating [45]. 

Many studies support the value of PPPs in improving nutritious food access and intake. A PPP was deployed through safety net clinics in Cuyahoga County, Ohio for patients experiencing hypertension and at risk of food insecurity. Participants received four $10 vouchers to use at a local farmers market. As a result of this intervention, one third of patients reported attending a farmers market for the first time, improved dietary behavior, and increased communication with their healthcare provider [46]. 

Another PPP was implemented at an academic healthcare system through a Food as Medicine clinic, wherein food-insecure participants received an allotted amount of food for up to one year. While short-term health outcomes did not improve significantly, self-reported dietary behaviors did, suggesting some degree of efficacy of the program, with helpful directives for future research, such as larger sample sizes, longer term studies, and tracking of additional health metrics [47].

A pilot PPP in Harris County, Texas provided 172 eligible food insecure adults 30 pounds of fresh produce at a food pantry up to 12 times over a 6-month period. This program was effective in significantly reducing community food insecurity while ensuring increased intake of health-promoting fruits and vegetables [48].

Another food insecurity PPP based in Washington, DC enrolled 25 eligible families in a 12-month at-home food delivery and virtual nutrition education intervention. This mixed methods study identified the feasibility of a home-delivery-based food prescription program. Results indicated an increased intake of fruits and vegetables in some children, but not all. Study findings suggested a non-significant reduction in food insecurity, which reveals an area for future research [49].

These studies reveal that PPPs are an effective way to promote increased nutrient intake, most often through fruits and vegetables, especially for individuals or communities that face food or nutrition insecurity. Longer-term studies, increased funding, expanded nutrition education and counseling opportunities, assessment of factors that drive or stifle consumer food choice and behavior change, and identification of communities most in need of such programs can help drive the availability and effectiveness of PPPs forward.

#### 2.2.3. Teaching Kitchens

TKs are a subset of the broad FIM discussion. TKs are often described as “learning laboratories” that offer training and education in nutrition, culinary skills, mindfulness, physical activity, and motivational interviewing [50]. TKs equip individuals with the skills and confidence to prepare nourishing and delicious meals and enjoy them mindfully. TKs can be utilized by a variety of audiences, including undergraduate students (especially those in nutrition and/or dietetic programs), graduate students, medical students, residents, fellows, physicians, nurses, employees, organization leaders, and community members. Classes offered in TKs are typically facilitated by physicians, dietitians, chefs, or a combination. There are many ways in which a TK can be set up: virtually offered through video broadcast, as a mobile “pop up” site, or as a built-in facility at an institution or business. TKs are the setting in which culinary medicine, lifestyle medicine, integrative medicine, and conventional medicine can all be taught through the lens of food [51].

Many studies support the value of TKs in improving individuals’ behaviors and health outcomes. A first-of-its kind pilot study at the Culinary Institute of America in Hyde Park, New York enrolled 40 non-chef employees to participate in weekly, 2.5-h didactic lectures and culinary demonstrations and bi-monthly (two times per month), 5-h hands-on culinary lessons (which included a “mindful lunch”) for a period of 14 to 16 weeks. Participants were also provided with complimentary gym access and connected with a certified health coach for weekly phone call check-ins. Participants experienced statistically significant (*p* < 0.05) decreases in body weight, BMI, waist circumference, systolic and diastolic blood pressure, and total cholesterol after 14 to 16 weeks. At 12 months, reductions in diastolic blood pressure and waist circumference remained statistically significant (*p* < 0.05). This intervention, which did not prescribe a restrictive diet, but rather didactic education, experiential learning, and health coaching, supports the idea that a multidisciplinary approach—one component of which can include a TK—should be considered when encouraging long-term health and lifestyle changes [52].

Another study conducted at Emory University’s Healthy Kitchen Collaborative enrolled 38 healthcare and university employees in a 10-week TK program. This study assessed changes in participants’ micronutrient intake based on Dietary Reference Intake guidelines, as well as diet quality using the Healthy Eating Index. Although changes in micronutrient intake were not statistically significant, researchers did find the 18% increase in the number of participants meeting high micronutrient adequacy to be clinically meaningful. A statistically significant increase in seafood and plant proteins was observed after 3 months, and clinically meaningful increases in vegetables, greens and beans, total fruit, whole fruit, whole grains, total dairy, total protein, and fatty acids, as well as decreases in refined grains and added sugars, were also observed [53].

A multicenter project across 32 medical schools and residency programs enrolled 4125 medical trainees in the Cooking for Health Optimization with Patients-Medical Trainees (CHOP-MT) program. A total of 1219 participants received instruction in a 32-h hands-on course, which was divided into eight 4-h modules delivered once per week for eight weeks. These modules exposed trainees to case-based learning, hands-on cooking sessions, and nutrition counseling skill development. A total of 2906 participants received traditional medical nutrition education and no hands-on course instruction. Participants in the intervention group were significantly more likely to report increased adherence to Mediterranean dietary principles, as well as competence regarding these principles compared to the control group. Participants were also more than two times as likely to agree that nutrition counseling should be routine in direct patient care. This study supports the idea that TKs in the context of medical training has the potential to positively impact trainees’ individual dietary patterns, as well as those of their future patients [54].

Given the efficacy of both PPPs and TKs separately, there exists an opportunity to offer them jointly in order to increase the efficacy of FIM interventions. In addition to increased access to fresh produce, enhanced knowledge about how to prepare and incorporate these foods into dietary patterns could boost consumption and demand [52]. Further, PPPs offer the unique opportunity to use local agricultural resources, thereby closing the loop of local agriculture to TKs, theoretically leading to improved human and planetary health outcomes.

### 2.3. Consumer Food Choice: Influences and Opportunities

The main objective of TKs is to improve participants’ adoption of healthy behaviors. A person that follows a standard three-meals-per-day dietary pattern (recognizing that this is not the case for every individual) consumes 1095 meals per year. With every meal comes the opportunity to prioritize both physical and planetary health. However, most people are not motivated by these factors. Instead, food choices are influenced by palatability, culture, cost, availability, convenience, habits, genetics, and environmental cues [55]. Yet these factors do not necessarily have to be at odds with one another. TKs offer a setting in which many different motivations for dietary behavior changes can be addressed. Those that work at the intersection of nutrition, health, and cooking agree that a planetary health diet can be simultaneously delicious, convenient, and affordable, but it undoubtedly takes education and hands-on skill development to recognize and apply this. In order to make planetary health diets accessible, individuals need the time, skills, energy, resources, and motivation to implement such dietary changes.

One strategy for promoting successful shifts in dietary behavior is addressing concerns associated with costs [56]. Highly-palatable calories offered in the form of UPFs tend to be inexpensive, whereas nutrient-dense foods, especially fresh fruits and vegetables, tend to be more expensive. Currently, 41.9% of the global population cannot afford a healthy diet [6]. With price as a major barrier for almost half of Americans, reliance on an industrialized global food system leaves individuals vulnerable to price inflation and supply chain challenges [57]. In order to initiate a shift toward planetary health diets, FIM programs must consider cost, convenience, and taste among the key drivers of food choice and consumption patterns [56,58]. 

TKs provide an opportunity to teach people how to adopt budget-friendly diets through both affordable ingredient use and culinary skill development. In this setting, instructors can educate people how to read and interpret various “best by” dates, thereby helping to reduce unnecessary disposal of foods that would otherwise be acceptable for consumption. Additionally, TKs provide the setting in which individuals can learn how to purchase appropriate quantities of food, repurpose ingredients, appreciate cosmetically imperfect produce, use less-desirable parts of certain foods such as celery leaves or broccoli stalks, make homemade stock from vegetable scraps, compost, preserve ingredients, and freeze leftovers. Developing these skills can provide both financial and environmental benefits. The less food a person wastes, the more money they are not “throwing away”. Further, at both the micro and macro scale, food waste contributes a significant amount of greenhouse gas emissions to the environment [59].

Encouraging consumption of seasonal produce is another way to help consumers reduce spending on fresh produce. One study demonstrated that peak-season produce prices were significantly lower than out of season produce prices at direct retail outlets [60]. As an example, strawberries can be up to 100% more expensive in December than they are in the spring [61]. Some local, in-season produce may also be more nutrient-dense. Consuming nutrient-dense foods is associated with a modestly decreased risk of cardiovascular disease, diabetes, and all-cause mortality [31]. An additional benefit of in-season produce is improved flavor, which may further encourage consumption [62].

In order to reap the benefits of nutrient-rich foods from local agriculture, consumers need to be equipped with the knowledge to turn whole food ingredients into delicious and easy-to-prepare meals in relatively short amounts of time and for a low cost. A survey conducted at the Cleveland Clinic identified that lack of time to prepare healthy meals and unfamiliarity with healthy cooking methods were two of the biggest barriers to healthy eating for American adults [57]. This demonstrates the need for enhanced culinary nutrition education for the public. Culinary techniques such as food preservation and storage may be especially important for those living in regions with shorter growing seasons or who are unable to access fresh food frequently. By working in partnership with local agriculture, TKs provide an excellent opportunity to help individuals overcome these barriers and enhance dietary diversity and quality through the utilization of seasonal, local foods. Nutrition educators and chefs at TKs can collaborate with farmers and producers to lead local initiatives to create seasonal menus, lessons, promotional materials, and food guides [63].

Some individuals that are not concerned with health or their food budgets may be motivated to shift their food habits after learning about the food system’s impact on the environment [64]. One study developed an interactive web-based intervention to encourage the uptake of environmentally friendly eating behaviors among university students and found that there was a significant increase in sustainable eating behaviors when compared to the control group [65]. Increasing awareness of sustainable diets in nutrition education settings will be important for planetary health and serves as a novel way to influence consumer choice.

#### Food Access and Nutrition Security

For low-income communities in particular, it can be very difficult to obtain nutritious foods. A study of a low-income neighborhood in Pomona, California found that 41% of food pantry clients did not live within walking distance of a store with a variety of fresh produce and 13% did not have access to any type of store with fresh produce [66]. This problem is widespread across the US and highlights that many Americans are unable to meet recommended nutrition guidelines due to existing environmental barriers and a lack of community resources [14].

The lack of available produce in combination with poor access to grocery stores can hinder healthy choices in resource-poor communities. On the contrary, community-supported agriculture programs offer flexible, cost-effective solutions that can provide a variety of seasonal products to local markets and individuals [14]. Programs like Farm Fresh Foods for Healthy Kids can improve food access and offer convenient pick-up options for participants, benefiting both families and local farmers [14,67]. A study in Eastern North Carolina revealed that consumers were attracted to purchase at a farmers’ market when it was closer to their residence and when cost savings and produce availability were greater than those at the supermarket [68].

The significance of local, seasonal, and affordable produce from farmers’ markets in driving behavioral change is evident [14,69]. Certain fruits and vegetables that are cultivated through sustainable farming practices and distributed through local channels possess better nutrient-to-cost ratios, indicating the potential to enhance nutrient density without significantly increasing consumer expenses [31,70,71,72]. 

Further, local food networks serve as avenues for community health education. Demonstrations of farming practices paired with healthy eating workshops can promote food as preventive medicine. In one interview, a Baltimore farmer explained how she highlights the diversity of the food she grows and teaches workshop participants how to utilize these foods in their kitchens. By doing so, she hopes to help families create lifelong healthy eating habits [10]. This example underscores the potential synergy between TKs, local agriculture, and community health education. Increasing access to affordable, local food options in combination with comprehensive culinary education could theoretically help reduce food/nutrition insecurity and chronic disease risks.

It is important to note that local food system movements across the United States, while certainly beneficial, have historically excluded low-income consumers and marginalized communities [73]. Given existing economic and food access disparities, it is important to support alternative food networks that focus on food sovereignty as a means of overcoming inequities to enable sustainable health habits. Food sovereignty is defined as “the right of peoples to healthy and culturally appropriate food produced through ecologically sound and sustainable methods, and their right to define their own food and agriculture systems” [74]. Food sovereignty movements have been linked to positive community health outcomes through greater social and environmental engagement, increased access to healthy food, enhanced nutrient density, the establishment of more resilient food systems, and the promotion of social justice and equity. Sourcing from and collaborating with local agricultural businesses with a commitment to food sovereignty is critical in integrating culturally relevant food and farming practices in nutrition intervention programs. 

Generally, health interventions have taken a one-size-fits-all approach, favoring the consumption patterns and lifestyles of certain cultures over others. A study on the impacts of culturally tailored nutrition education for Hispanic mothers found that adding cultural aspects such as country-of-origin native foods and recipes was associated with improvements in diet quality [75]. Individuals reported greater interest in trying new recipes and overall increases in daily fruit and vegetable consumption. Because eating behaviors are a way to express one’s cultural identity, it is imperative to design nutrition interventions with a group’s cultural orientation in consideration [76]. In supporting food justice movements, TKs can go beyond individual consumption patterns and play a role in addressing health inequities [73]. 

### 2.4. Cross-Disciplinary Collaboration Opportunities for Farmers, Chefs, and Healthcare Practitioners

Farmers grow food, retailers sell it, chefs prepare it, healthcare professionals prescribe it, and institutions fund it. When these activities are done in silos, the potential to drive revolutionary system changes is squandered. However, if and when these stakeholders join forces, there exists great potential to advance the FIM movement and bring about positive change for community well-being and environmental sustainability. The strategic alignment between farmers, chefs, and healthcare institutions can foster a resilient and interconnected food system that addresses challenges related to food insecurity, nutrition quality, and climate change [17]. Additional stakeholders including the food industry, food retailers, policymakers, scientists, and others also have opportunities to collaborate with farmers, chefs, and healthcare systems, but the role of each of these actors exists outside the scope of this paper.

#### 2.4.1. Farmers

In the existing agricultural system, “success” is commonly assessed through production measures such as yield. However, beyond the surface-level goal of cultivating crops and raising livestock for consumption and raw materials, the true essence of agriculture lies in nurturing the well-being of individuals [77]. Consequently, a farmer’s role extends beyond mere food production and into the provision of high-quality, nutrient-rich sustenance essential for promoting healthy lives. Farmers that collaborate with healthcare providers, community organizations, and food assistance programs can help ensure that fresh, locally sourced, and wholesome produce is accessible to individuals seeking dietary interventions for improved health outcomes. This alternate perspective underscores the crucial importance of farmers within modern healthcare systems.

Farmers possess the expertise and tools necessary to help transform the food system into one that protects human and planetary health. Healthy food, which underpins the preventative healthcare approach, can only be as healthy as the soil in which it is grown. With their intimate knowledge of the land, farmers have the ability to restore soil health, biodiversity, and climate resilience, which is critical in supporting community nutrition security [78,79]. By adopting regenerative and health-oriented agricultural practices, farmers can contribute to preventive healthcare on a large scale [4].

Within the FIM movement, farmers play a crucial role as contributors to public health. Their cultivation practices directly influence the nutritional content and quality of the produce that forms the foundation of this movement. Embracing agroecological and regenerative approaches that minimize the use of synthetic inputs, promote biodiversity, and prioritize soil health can lead to higher nutritional content in crops and enhanced microbial diversity, aligning with the movement’s objective of utilizing food to prevent and manage health conditions [37,80,81,82,83].

The relationship between farmers and TKs is symbiotic. Small and medium-scale farmers often face financial hardships which can be especially difficult for those practicing or looking to practice regenerative agriculture based on current federal farm policy that favor industrial methods [84]. However, TKs offer a long-term collaboration opportunity that could provide farmers with a sustainable income source. By embedding relationships with local producers into the TK structure, farmers may be incentivized to grow food that is both nourishing and sustainable. These relationships are beneficial to not only farmers and TKs, but to the community as a whole.

TKs provide a platform for farmers to showcase their fresh, seasonal produce and educate participants about the nutritional and environmental benefits of their ingredients. Farmers’ involvement in TKs may also provide an added layer of expertise and authenticity to culinary education. Their direct connection to the land and the food they cultivate enables them to share knowledge about ingredient selection, seasonality, quality, nutrition, and flavor. This collaboration can enrich participants’ understanding of sustainable sourcing and enhance their appreciation of the flavors and nutritional benefits of locally grown foods. TKs may also increase consumers’ demand for such foods, thus supporting the continual production of nutritious food. Farmers’ presence can foster a deeper connection between the food on the plate and its origins, creating a holistic experience that extends beyond the kitchen into a broader awareness of nutrition and food systems.

#### 2.4.2. Chefs

The role of the chef has undergone significant transformation in recent decades. Increasingly, chefs are recognized for their potential to positively impact public health and environmental sustainability. This is exemplified in farm-to-table restaurant concepts, plant-forward menus, and chefs’ involvement in TKs and other FIM or culinary medicine programs [17,85,86]. Chefs play a crucial role in facilitating these teaching environments, leveraging their culinary expertise to inspire healthier cooking practices and dietary choices [87,88]. By emphasizing the utilization of locally sourced ingredients, chefs can foster a direct link between the community and local agricultural producers. Further, as advocates for responsible sourcing, chefs can prioritize seasonal and locally-available produce, thereby reducing the carbon footprint associated with food transportation and distribution.

Organizations such as the UNFAO, EAT-Lancet Commission, and the World Wildlife Fund are encouraging rapid shifts in food production and consumption in response to a widespread understanding of the global food system’s impact on climate change and environmental degradation. Given their role as intermediaries between farmers and consumers and as cultural trendsetters, chefs are at the forefront of food system transformation. As a result, chefs can drive demand for new diets that are both sustainable and healthy [89]. Sustainable gastronomy is a growing movement which “takes into account where the ingredients are from, how the food is grown and how it gets to our markets and eventually to our plates” [90]. Using recipes inspired by local food influences, flavors, and biodiversity, chefs can model that sustainable gastronomy can both taste good and support health, thereby encouraging shifts in consumers’ habits [89]. Chefs can use their platforms to teach communities how to prepare dishes that reduce food waste, are plant based, utilize nutritious ingredients, and taste delicious, catalyzing a dialogue that empowers communities to transform food systems through consumers’ own kitchens.

The Chef Initiative was a two-year pilot project led by the Harvard School of Public Health, Project Bread, Boston Public Schools, and the Boston Public Health Commission. The program was conducted in two Boston middle schools that brought in a chef to develop recipes, plan menus, and train cafeteria staff two or three days per week. Two other schools were selected as the control group and served usual school cafeteria food. The chef designed menus similar to the traditional Boston Public School menus but incorporated more whole grains, fruits, vegetables, and flavor, and less sugar, salt, saturated fat, and trans fat. At the Chef Initiative schools, students consumed on average two more servings of vegetables per week. Additionally, 86% of students at the Chef Initiative schools chose at least one serving of whole grains, compared to 35% of students in the control group [87]. This pilot project demonstrated the powerful effect culinary techniques can have on influencing consumer food choice and behavior change.

Chefs are instrumental in the FIM movement and especially TKs. Given that taste is a key determinant of food choice, chefs can help translate nutritional knowledge into practical and appealing culinary creations, ensuring that therapeutic foods are not only healthy but also enjoyable [32,91]. Applying nutrition principles to food preparation transforms learning into a delicious, nutritious experience that allows people to see, feel, and taste what nutrition is all about [92,93].

As FIM, TKs, local agriculture systems, and planetary health diets continue to gain momentum, chefs will be key actors in transforming the modern food landscape. By embracing opportunities beyond the restaurant setting, chefs can leverage their culinary expertise to foster healthier communities, promote sustainability, and advocate for holistic approaches to nutrition.

## 3. Case Studies

While the intersection of TKs and agriculture are relatively new in the academic setting, innovative organizations have already been forging ahead to close the gaps between individual and planetary health. These innovations take the form of chef-centered hospital food service redesign, hospital rooftop gardens, community- and hospital-based TKs working in connection with farms, and pop-up/mobile TKs at local farms, school gardens, and community gardens.

Traditional hospital food has long born a subpar reputation due to a combination of factors rooted in practicality, budget constraints, and specific dietary considerations. The focus in hospital food service is often on adhering to dietary guidelines, which can overshadow culinary finesse. Hospital administrators recognize that food is a factor in overall patient satisfaction, which has led to an increased awareness of the importance of providing appealing and nutritious meals in healthcare settings. Some hospitals are investing in upgrading their food service operations, hiring skilled chefs and focusing on fresh, locally sourced ingredients to improve the overall dining experience for patients and visitors.

### 3.1. Northwell Health

Northwell Health, located in New York, New York, is the model for transforming a hospital food system, trailblazing how this might be accomplished across the country. In 2017, Northwell hired a Michelin-star chef to oversee the complete reinvention of its hospital food service programs. He leaned into his vast network of chefs and encouraged them to use their experience and creativity to redesign Northwell’s menus to prioritize both flavor and patient health. Thus far, they have demonstrated that feeding patients in this manner does not significantly increase food costs, and dramatically improves patient satisfaction [94]. In 2022, they partnered with the Queens County Farm Museum to utilize the farm’s food in hospital menu items, as well as to lead free, community-based cooking demonstrations in their “farm-to-table wellness program”. This forward-thinking partnership represents the opportunity for hospital systems to position themselves within communities as leaders of both human and planetary health [95].

By working with local agriculture, prioritizing FIM in patient feeding, preparing dishes that support planetary health, conducting community culinary demonstrations, influencing consumer food choice, supporting healthy food access, and promoting cross-disciplinary collaboration between chefs, farmers, and healthcare administrators and practitioners, Northwell is the true leader in healthcare and agricultural systems changes. Future opportunities to continue supporting individual and environmental health may include utilizing a full TK model for patients and/or providers, integrating hydroponic growing systems within future facilities, expanding FIM or PPP opportunities for patients, and identifying and addressing gaps between hospital-provided food and at-home consumer choices.

### 3.2. Turner Farm and the Osher Center for Integrative Health at the University of Cincinnati College of Medicine

Turner Farm and Meshwa Farms are adjoined organic farms in Indian Hill, Ohio, covering 230 acres of permanently protected land. Turner Farm renovated their 100-year-old working barn into an on-site TK that utilizes ingredients grown on the farm and by other local producers. In this setting, chef educators teach community members, as well as patients and medical students from the University of Cincinnati College of Medicine, about organic food production as well as essential skills for preparing flavorful and nourishing meals [96,97].

This collaboration represents a successful integration of sustainable agriculture with fully functional TK programs, and is offered both virtually and in-person. Future opportunities to continue supporting individual and environmental health may include integrating foods grown on the farms into patient feeding, leading more free community-based culinary demonstrations, using farm produce in hospital-based food pantries or PPPs, and identifying and addressing gaps between TK classes and consumer food choices.

### 3.3. Boston Medical Center

Boston Medical Center (BMC) operates a 2658-square foot rooftop farm atop their main medical facility in Boston, Massachusetts. Food grown on the farm is used in the hospital’s food pantry, TK, and food service operations. During operational months (April to November), 5% of the food that is distributed to food-insecure patients through the food pantry is provided by the farm. Produce is also used in BMC’s TK, where culinary and nutrition instructors lead classes for community members, as well as for medical students and physicians [98].

The rooftop farm helps meet the needs of the community by alleviating food insecurity, promoting environmental sustainability, and fostering engagement, education, and well-being for employees, patients, and the community. This framework holds potential for replication by hospitals across the country. Future opportunities to continue supporting individual and environmental health may include leading more free community-based culinary demonstrations, partnering with other farms or institutions to increase available produce, improving patient-feeding menus, expanding FIM and PPP efforts, and identifying and addressing gaps between TK classes and consumer food choices.

### 3.4. The Charlie Cart Project

The Charlie Cart Project was established in 2015 to use pop-up mobile kitchens and food education curriculum to empower children to make lifelong healthy choices. This initiative aims to bridge connections between food, well-being, and the environment to set kids up for a lifetime of health. Charlie Carts can be found across the nation in K-12 schools, farmers markets, libraries, hospitals, food banks, and more. Due to their portable nature, Charlie Carts have the potential to both bring the farm to the kitchen by using locally sourced food, and the kitchen to the farm by leading demonstrations in collaboration with local farmers [99].

This organization is helping drive consumer behavior change from a young age through a TK model that brings healthy food demonstrations to young students. Future opportunities to continue supporting individual and environmental health may include working with more local farmers, expanding educational programming to older audiences, working in collaboration with PPPs at pick-up sites, and identifying and addressing gaps between TK classes and long-term consumer food choices and health outcomes.

### 3.5. Harlem Grown

Harlem Grown was established in 2011 “to inspire youth to lead healthy and ambitious lives through mentorship and hands-on education in urban farming, sustainability, and nutrition” [100]. Through their 13 urban farms, they lead programs that educate and mentor children about food, the environment, culture, community, and health. Their mobile TK program encourages participants to try new foods, develop cooking skills, discover culinary traditions, expand their creativity, and learn about nutrition.

Integrating urban agriculture and culinary skills with early childhood education supports healthy food access, influences consumer food choice, encourages local food production, and promotes cross-disciplinary collaboration between chefs, farmers, and community members. Future opportunities to continue supporting individual and environmental health may include expanding the mobile TK curricula, increasing the number of operational urban farms, partnering with healthcare or school systems, working in collaboration with PPPs at pick-up sites, and identifying and addressing gaps between TK classes and/or agriculture exposure and long-term consumer food choices and health outcomes.

These case studies point to the value and feasibility of cross-disciplinary collaboration in bridging the gap between TKs and local agriculture. Further, they suggest that future wellness care ensembles can and should include healthcare administrators and practitioners, farmers, and chefs in order to promote access to and uptake of planetary health diets.

## 4. Discussion

Given the exciting momentum around FIM, TKs, local agriculture, and sustainable gastronomy, there exists great potential to align the intentions and efforts of farmers, chefs, and healthcare practitioners to improve human and planetary health. Creativity and collaboration will be required in order to build a future in which planetary health diets are not only possible, but also mainstream. First of its kind, innovative, equity-centered policy interventions, healthcare and school facilities, research projects, agricultural systems, and educational programs must be deployed broadly in order to make this possible. Activities may include building vertical and/or hydroponic farms in hospitals and schools, creating rooftop gardens on healthcare or university facilities, transforming hospital or school cafeterias into TKs, connecting healthcare systems with farms and farmers markets, incorporating hands-on nutrition education into school curricula from kindergarten through continuing education, enriching government food programs to incorporate TKs with local agriculture movements, and broadening the FIM movement to prioritize local agriculture and access to TKs in their many forms. Together, these activities can help improve nutrition security, nutrient density, health outcomes, community well-being, and planetary health.

Potential challenges that may arise when attempting to integrate such interventions include identifying and accessing funding, aligning institutional leadership goals with program objectives, influencing food policy to further promote human and planetary health, hiring and retaining personnel to operate TK programs, establishing partnerships with local agriculture programs, recruiting and retaining consumer participation, and evaluating both short- and long-term effectiveness of TK and agriculture programs. However, these challenges should not be viewed as threats to positive change, but rather opportunities to establish unexpected collaborations between private- and public-sector actors.

## 5. Conclusions

The prevailing issue of escalating diet-related chronic diseases amidst a compromised food system necessitates a reevaluation of the traditional approach. The historical trajectory of industrialized agriculture has led to detrimental consequences, compromising nutrition, disconnecting individuals from their food sources, and aggravating climate change. Amidst this backdrop, a substantial portion of the population remains unable to access nourishing diets. The call to action is clear: a paradigm shift is essential. 

Integrating healthcare and agriculture systems presents an opportunity to address these concerns holistically. By forging novel connections between healthcare institutions and local agriculture, such as TKs in collaboration with diverse farming initiatives, a promising path emerges. In addition to health practitioners, the FIM movement must also include those who are growing the food—farmers—and those who are preparing it—chefs. This synergy could not only counteract the climate crisis and improve health outcomes, but also foster community resilience and interdisciplinary cooperation. Now is the time to champion the approach that envisions a harmonious coexistence of agriculture and healthcare, nurturing both human well-being and planetary health.

## Data Availability

Not applicable.
